# The Processing of the Second Syllable in Recognizing Chinese Disyllabic Spoken Words: Evidence From Eye Tracking

**DOI:** 10.3389/fpsyg.2021.681337

**Published:** 2021-10-27

**Authors:** Youxi Wang, Xuelian Zang, Hua Zhang, Wei Shen

**Affiliations:** ^1^Center for Cognition and Brain Disorders, The Affiliated Hospital of Hangzhou Normal University, Hangzhou, China; ^2^Institute of Psychological Science, Hangzhou Normal University, Hangzhou, China; ^3^Zhejiang Key Laboratory for Research in Assessment of Cognitive Impairments, Hangzhou, China

**Keywords:** second syllable, Chinese, spoken word recognition, eye tracking, printed-word paradigm

## Abstract

In the current study, two experiments were conducted to investigate the processing of the second syllable (which was considered as the rhyme at the word level) during Chinese disyllabic spoken word recognition using a printed-word paradigm. In Experiment 1, participants heard a spoken target word and were simultaneously presented with a visual display of four printed words: a target word, a phonological competitor, and two unrelated distractors. The phonological competitors were manipulated to share either full phonemic overlap of the second syllable with targets (the syllabic overlap condition; e.g., *小篆*, *xiao3zhuan4*, “calligraphy” vs. *公转*, *gong1zhuan4*, “revolution”) or the initial phonemic overlap of the second syllable (the sub-syllabic overlap condition; e.g., *圆柱*, *yuan2zhu4*, “cylinder” vs. *公转*, *gong1zhuan4*, “revolution”) with targets. Participants were asked to select the target words and their eye movements were simultaneously recorded. The results did not show any phonological competition effect in either the syllabic overlap condition or the sub-syllabic overlap condition. In Experiment 2, to maximize the likelihood of observing the phonological competition effect, a target-absent version of the printed-word paradigm was adopted, in which target words were removed from the visual display. The results of Experiment 2 showed significant phonological competition effects in both conditions, i.e., more fixations were made to the phonological competitors than to the distractors. Moreover, the phonological competition effect was found to be larger in the syllabic overlap condition than in the sub-syllabic overlap condition. These findings shed light on the effect of the second syllable competition at the word level during spoken word recognition and, more importantly, showed that the initial phonemes of the second syllable at the syllabic level are also accessed during Chinese disyllabic spoken word recognition.

## Introduction

Humans can understand speech quickly and almost without effort. Successful speech comprehension requires the segmentation of the continuous speech stream into discrete spoken words, and the mapping of spoken words onto the corresponding lexical representations in the mental lexicon. After decades of research, current spoken word recognition models have reached a common consent that a set of phonologically similar word candidates compete for activation as the speech signal unfolds. With the incremental availability of more disambiguating phonemic information, candidates that no longer match the speech signal are inhibited while the target word is activated until it finally wins the competition. However, current spoken word recognition models debate the size of the competitor set and the time point at which word competition occurs. For example, the cohort model (Marslen-Wilson and Tyler, 1980) posits that only competitors sharing similar word-initial phonemes with targets (e.g., “cloud” vs. “clothes”) are activated for competition in the earlier phrase of speech perception. Other types of phonologically similar words such as rhymes (i.e., words that share ending phonemes such as “cloud” vs. “proud”) are not involved in the competition process. By contrast, the Neighbor Activation Model (NAM; Luce, 1986; Luce and Pisoni, 1998) and the TRACE model (McClelland and Elman, 1986) allow for competition among a much broader set of candidates such as rhymes and other phonological neighbors. The NAM proposes that word candidates that differ in no more than one phoneme are neighbors competing for recognition. But the hypothesis regarding the speech temporary is not considered in the NAM model. The TRACE model assumes that word recognition is a continuous mapping process in which competitors are activated continuously. The TRACE model makes an explicit assumption that cohorts and rhymes both participate and compete in the process of spoken word recognition. Moreover, TRACE also predicts that the cohort competitors are activated strongly and earlier while rhyme competitors are activated weakly and later due to the temporal properties of speech information.

Although a growing number of studies have provided compelling evidence for the existence of cohort competition during spoken word recognition (Marslen-Wilson and Zwitserlood, 1989; Allopenna et al., 1998; Desroches et al., 2006, 2009; Simmons and Magnuson, 2018), the rhyme competition effect has been found to be weak and less reliably detected than the cohort effect in alphabetical languages like English (Allopenna et al., 1998; Simmons and Magnuson, 2018; Hendrickson et al., 2020) and elusive in non-alphabetical languages such as Chinese (Liu et al., 2006; Malins and Joanisse, 2010; Zhao et al., 2011). The aim of the current study is to investigate the role of rhyme (i.e., second syllable) in Chinese spoken disyllabic word recognition. Specifically, we aimed to examine whether rhyme competition is actually involved, and more importantly, whether the initial phonemes of the second syllable, is activated during Chinese disyllabic spoken word recognition.

Existing evidence from eye-tracking studies has demonstrated that rhymes compete in spoken word recognition but the rhyme competition effect is weaker and less stable than the cohort competition effect in alphabetical languages (Allopenna et al., 1998; Desroches et al., 2006, 2009; Brouwer and Bradlow, 2016; Simmons and Magnuson, 2018; Hendrickson et al., 2020). In the seminal visual-world paradigm study conducted by Allopenna et al. (1998), participants were presented with a visual display of four objects: a target (e.g., a beaker), a cohort competitor (e.g., a beetle), a rhyme competitor (e.g., a speaker) and an unrelated distractor (e.g., a carriage). Participants followed a spoken instruction (e.g., “please pick up the beaker”) as eye movements were recorded. Results of the fixation probability showed more fixations on the cohort and rhyme competitors than on the distractors. More importantly, the cohort competition effect was found to be stronger and occurred earlier than the rhyme competition effect. Similarly, another eye-tracking study generalized the cohort and rhyme competition effect from adults to normally developing children (Desroches et al., 2006). However, the comparison of the normal group and a dyslexic group of children showed that both groups exhibited a significant cohort competition effect (i.e., cohort competitors attracted more fixations than the baseline condition), but only the normal group directed more visual attention to the rhyme competitors (e.g., sandal) than to the unrelated distractor when recognizing target words (e.g., candle), while such a rhyme competition effect was absent in the dyslexic children. This finding suggests that the rhyme competition effect is less likely to be observed than the cohort effect and is more vulnerable to other factors such as the phonological deficits of the dyslexic children. Further evidence for the rhyme competition effect was provided by Event-Related Potential (ERPs) studies. For example, Desroches et al. (2009) used ERPs to examine the time course of phonological competition in English spoken word recognition. In the picture-word matching task, participants were required to judge whether a spoken word matched a visual picture. They found that both cohort competitors (e.g., CONE vs. comb) and rhyme competitors (e.g., CONE vs. bone) elicited significantly larger N400 effects than did the complete match condition (e.g., CONE vs. cone). In addition, results from the *post hoc* analysis showed that the N400 effect in the rhyme condition was much weaker than that in the cohort condition. Together, the above findings are in line with the assumption of the TRACE model that rhymes compete later and much more weakly than do cohorts.

While the above-mentioned studies proposed that rhymes do compete for recognition in alphabetical languages, the role of rhymes in Chinese spoken word perception is rather elusive, given that some studies found rhyme competition effect while other did not. For example, in an eye-tracking study, Meng (2014) investigated rhyme competition in disyllabic Chinese words by manipulating rhyme competitors sharing entire phonemes of the second syllable in an eye-tracking study. Participants were presented with spoken target words (e.g., *桥洞*, *qiao2dong4*, “arches”) in spoken sentences and were simultaneously presented with a visual display of four words: a cohort competitor (e.g., *樵夫*, *qiao2fu1*, “woodman”), a rhyme competitor (e.g., *果冻*, *guo3dong4*, “jelly”), a tonal competitor (e.g., *麻袋*, *ma2dai4*, “gunny-bag”) and an unrelated distractor. The results showed longer total viewing time on the cohort competitors than on distractors, suggesting the activation of cohort competitors. However, they did not find any interference effect induced by rhyme competitors and the total viewing times for rhymes were even shorter than those for the distractors. On the other hand, an ERP study conducted on disyllabic words by Liu et al. (2006) found evidence for rhyme activation. Liu et al. (2006) found that rhyme competitors (e.g., *水池*, *shui3chi2*, “water pool”; target words: *电池*, *dian4chi2*, “battery”) elicited earlier N400 amplitudes than did cohort competitors (e.g., *电路*, *dian4lu2*, “electric stove”; target words: *电池*, *dian4chi2*, “battery”). Moreover, the survival time of the N400 amplitude was much shorter for the rhyme competitor than for the unrelated condition. This indicates that the N400 effect elicited by the rhyme competitor disappeared earlier than that in the unrelated condition. These findings provide evidence for the activation of both cohort and rhyme information in Chinese, and the rhyme effect occurred later in the processing time course than did the cohort effect.

To summarize, evidence from previous studies for rhyme processing in Chinese remains elusive. We assumed that the inconsistent findings between Liu et al. (2006) and Meng (2014) could be due to that the degree of sentence constraints differs greatly across the two studies. Liu et al. (2006) examined rhyme processing in the context of highly predictive sentences (the mean predictability score was 6.33 on a 7-point scale), while the spoken sentences used in Meng (2014) were neutral ones. The predictive context contains highly constraining contextual cues for the upcoming words while the neutral context or the isolated word situation cannot provide any valid contextual cues. Studies have shown that phonological processing of words can be affected by the sentence predictability. For example, the lexical tone exhibits stronger constraining role during Chinese spoken word recognition in the predictive context compared to the neutral context or isolated word recognition; and a similar modulating role of lexical tone was found between the neutral context or isolated word recognition (Liu and Samuel, 2007; Shen et al., 2020). The mechanism underlying the prediction is assumed to be the pre-activation, that is, the activation occurs earlier before the presentation (Otten et al., 2007; Laszlo and Federmeier, 2009). It was found that both phonological/form and semantic information of words can be pre-activated in highly constraining contexts (Ito et al., 2016). Therefore, it is possible that the rhyme information was pre-activated in highly predictive sentences, which would result in a larger and earlier rhyme competition effect compared to the neutral context or isolated word recognition.

However, the neutral context or the isolation word recognition cannot fully account for the absence of the rhyme competition effect in Meng’s study given that the role of rhyme is consistently observed in the isolated word recognition in alphabetical languages (Allopenna et al., 1998; Desroches et al., 2006, 2009). We speculated that the absence of rhyme competition effect could be due to the inappropriate experimental design and the data analysis in Meng’s study. First, presenting more than one competitor in a same visual display (see the experimental manipulation in Meng, 2014) could lead to cognitive competition among different competitors and thus weaken the observed the rhyme competition effects. It has been shown that the competitor effect to be reduced and occurred later with the increasing number of visual referents (Sorensen and Bailey, 2007), which was explained by the working memory capacity limitation. The representations of visual referents would become faded in the working memory in the larger visual arrays compared to the small visual arrays (Sorensen and Bailey, 2007; Huettig et al., 2011). Second, the semantic plausibility between the spoken sentences and the rhyme competitors was not controlled. For example, the rhyme competitors (e.g., *果冻*, *guo3dong4*, “jelly”) were implausible for substitution into sentences like “*从这里穿出_*” (“Walking out from _”). A prior study found that the visual objects which were semantically plausible extension of the preceding context attracted more visual attention than the objects that were semantically implausible (Frassinelli and Keller, 2012). This is because the words in sentences can generate semantic properties concerning with the concept and such information can be used in allocating visual attention to the well-matched visual objects (Frassinelli and Keller, 2012). Therefore, the uncontrolled semantic plausibility could have resulted in fewer fixations on the rhyme competitors in Meng’s study. Third, no time course analysis of the rhyme processing was conducted in Meng (2014) and the indexes of fixation durations (*first fixation duration* and *total fixation duration*) and the *number of fixations* cannot necessarily reflect the rhyme competition occurred in the specific processing stage during spoken perception (e.g., a relatively later rhyme processing found in alphabetical languages; Allopenna et al., 1998; Desroches et al., 2006).

Based on the above debates, the current study aimed to address two research questions. First of all, we aimed to clarify whether a rhyme (i.e., the second syllable^1^) is activated for competition in disyllabic Chinese words using a visual world paradigm with eye tracking. Noted that Chinese syllables differ from alphabetical languages in the following aspects. First, Chinese syllable can refer to individual morphemes and can be used as mono-syllabic words (Zhou and Marslen-Wilson, 1994). This unique property enables that the rhymes (i.e., the second syllable in the disyllabic words) in Chinese are more salient than alphabetical languages like English. Second, Chinese syllables are composed of segmental information (i.e., the phonemes) and suprasegmental information (i.e., lexical tone). Thus, the syllable processing is more complicated than in alphabetical languages. Current spoken word recognition models (TRACE and NAM models) are proposed on the basis of the alphabetical languages and it remains less clear that the extent to which the rhyme information across languages is processed differently or similarly. Given the unique property and structure of the Chinese syllables, we assumed that the role of rhyme information in Chinese may be different from that in the alphabetical languages. In addition, our current study focused on word but not character processing is because disyllabic word plays a predominant role in Chinese (73% of words in Chinese are disyllabic words; Zhou and Marslen-Wilson, 1994) which is practically and theoretically more significant to examine the rhyme processing. In addition, the target words used in prior alphabetical studies are usually disyllabic words (Allopenna et al., 1998; Desroches et al., 2006; Simmons and Magnuson, 2018); thus, examining rhyme processing in the disyllabic Chinese words allows us to explore the similarities and differences of rhyme processing across between Chinese and English.

The second question of the current study was to examine whether an embedded cohort of the rhyme, that is initial phonemes of the second syllable in Chinese words, is activated for competition during spoken word recognition. Note that more than 73% of the Chinese words are disyllabic and each syllable in the disyllabic words can be used independently as a free monosyllabic word (Zhou and Marslen-Wilson, 1994). Therefore, the second syllable of the disyllabic words is not only the rhyme at the whole word level (Liu et al., 2006; Meng, 2014), but also contains its own initial and end parts at the monosyllabic level. For example, in the disyllabic word *“gong1zhuan4”* (*公转*, “revolution”), the second syllable “*zhuan4*” is the rhyme which contains monosyllabic-initial part “zhu-” and monosyllabic-end part “-uan.” The TRACE model assumes that cohort and rhyme within a word are accessed in temporal order and compete during spoken word recognition, but it makes no assumptions about whether there is also a further competition from the monosyllabic-initial/end. Chinese syllables are ideal material to examine this issue.

Regarding the processing of monosyllabic word, previous studies showed that the cohort part plays a more important role than the rhyme part. For example, in an eye-tracking study, Malins and Joanisse (2010) presented participants with a spoken Chinese monosyllabic word (e.g., *床*,^2^ *chuang2*, “bed”) and a visual display of four objects simultaneously: a target (e.g., *chuang2*, “bed”), a cohort competitor (e.g., *船, chuan2*, “ship”)/a rhyme competitor (e.g., *黄, huang2*, “yellow”) and two phonologically unrelated distractors. Participants were instructed to select the corresponding referent of the target in the visual display. The results showed a significant cohort competition effect with cohort competitor attracted more fixations than the distractors. In comparison, comparable fixations were found between the rhyme competitor and distractors, suggesting that rhyme information was not involved (as compared to cohort) in lexical competition during spoken word recognition. A similar pattern of stronger and sustained role of cohort competitor compared to the rhyme competitor was also observed in ERP studies (e.g., Malins and Joanisse, 2012). Given that the cohorts have more weight than the rhymes during spoken word processing, in this study, we investigated the issue that whether the embedded cohort of the rhyme (i.e., second syllable) could also be activated during spoken perception in Chinese.

In the following experiments, we adopted a printed-word paradigm with eye tracking to investigate the processing of the second syllable processing in Chinese spoken word perception (Huettig and McQueen, 2007; McQueen and Viebahn, 2007). The visual world paradigm has been widely used to explore phonological processing in visual word recognition (Shatzman and McQueen, 2006; Huettig and McQueen, 2007; McQueen and Viebahn, 2007; Weber et al., 2007; Ito et al., 2018; Shen et al., 2018). Compared to the ERP technique, the visual world paradigm with eye tracking has several advantages: (1) In ERPs studies, some explicit responses such as eye blinks and moving eyes would cause a great deal of electrical noise on the EEG signals (Rayner and Clifton, 2009), while the eye movements recording can occur implicitly without the interferences from explicit responses (Huettig and McQueen, 2007); (2) In ERP studies, incongruent spoken sentences are usually constructed when investigating the spoken comprehension (e.g., Liu et al., 2006), the eye movements recording can be recorded in a more natural language comprehension environment with the normal spoken sentence as stimuli; thus has higher ecological validity than the ERP technique. The printed-word paradigm adopted in the current study is a variation on the visual world paradigm in which the printed words replace visual pictures (McQueen and Viebahn, 2007). In addition, to avoid potential confounding that may harm the observation of rhyme competition effect, in the current study we adopted the following manipulations: (a) Only one type of phonological competitors (i.e., the rhyme competitor) was presented in each visual display. This could reduce the potential interference from any other competition effects. (b) Target words were presented in isolation (without sentence context) to avoid any possible prediction or semantic plausibility effect from sentence context; (c) A detailed time course analysis was conducted to tap into the rhyme competition in Chinese.

In the current study, participants viewed a display of printed words with simultaneous verbal presentation of target words. For a given target word, the competitors either shared the full phonemes of the second syllable with the targets (hereafter called “the syllabic overlap condition”), or shared partial (i.e., initial) phonemes of the second syllable (hereafter called “the sub-syllabic overlap condition”) with targets. The cohort model posits that rhyme information is not accessed during word recognition, and thus no competition effect should be observed in the syllabic overlap condition. On the other hand, both TRACE and NAM predict a significant phonological competition effect in the syllabic overlap condition, that was, a rhyme competition effect should be observed. In addition, based on the prior findings according to monosyllabic words that cohorts have more weight than the rhymes during spoken word recognition (Malins and Joanisse, 2010, 2012), we hypothesized that if the second syllable (i.e., rhyme) can be activated, the initial phonemes of the second syllable would also be activated to some extent.

Furthermore, the manipulation of two conditions also allows us to test the hypothesis of “phonological similarity.” According to the TRACE model, “global similarity” plays an important role in mapping spoken words onto lexical representations (McClelland and Elman, 1986). This assumption predicts that the degree of word activation varies with phonological similarity. Using the eye-tracking technique, Shen et al. (2018) manipulated the phonological similarity of the first syllable in Chinese disyllabic words and found that more fixations were allocated to high-similarity competitors (sharing full phonemic overlap with targets) compared to low-similarity competitors (sharing partial phonemic overlap with targets). If the mapping rule of “global similarity” also applies to second syllable processing in Chinese, we would observe a larger phonological competition effect in the syllabic overlap condition while a small effect in the sub-syllabic overlap condition.

## Experiment 1

### Method

#### Participants

Forty undergraduates (13 men, 27 women) from Hangzhou Normal University were randomly recruited and participated in the experiment. Their ages ranged from 18 to 27 years (*Mean age* = 21.2 years) and they were all native Mandarin Chinese speakers who had normal or corrected-to-normal vision and normal hearing. A monetary compensation was paid to each participant after the experiment. The research protocol reported here was approved by the ethics committee of the Institute of Psychological Sciences from Hangzhou Normal University.

#### Materials and Design

All spoken target words were recorded by a native Chinese female speaker at a normal speaking speed on the software Praat at a sampling rate of 44.1 kHz. All spoken target words were embedded in a spoken instructional carrier, “*请点击*” (“please click on”), and presented to participants through headphones.

**FIGURE 1:**
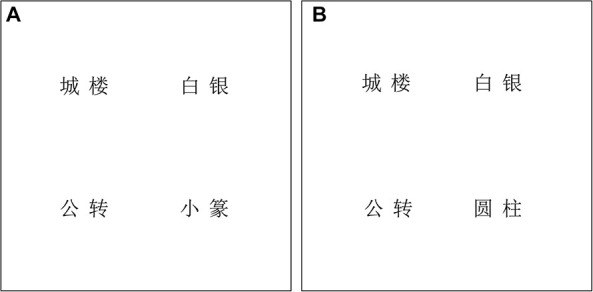
An example of a printed-word display in Experiment 1. For the spoken target word *公转*, *gong1zhuan4*, “revolution,” the printed-word display consisted of an identical target word, a phonological competitor word in the syllabic overlap condition **(A)** *小篆*, *xiao3zhuan4*, “calligraphy” or in the sub-syllabic overlap condition **(B)** *圆柱*, *yuan2zhu4*, “cylinder” and two unrelated distractors *城楼*, *cheng2lou2*, “gate tower” and *白银*, *bai2yin2*, “silver” in four corners of the display.

Fifty-four Chinese disyllabic words were selected as target items. Each visual display included a target word, a phonological competitor, and two distractors that were neither semantically nor phonologically related with target word. For each target word, there were two types of corresponding phonological competitors: a syllabic overlap competitor and a sub-syllabic overlap competitor. For the syllabic overlap condition, the phonological competitor shared all phonemes of the second syllable with the target words (e.g., *“小篆,” xiao3zhuan4*, “calligraphy” vs. *“公转,” gong1zhuan4*, “revolution”). For the sub-syllabic overlap condition, the competitor shared cohort-part phonemes of the second syllable with targets (e.g., *“圆柱,” yuan2zhu4*, “cylinder” vs. *“公转,” gong1zhuan4*, “revolution”). To maximize the possibility of observing the phonological competition effect, all phonological competitors were matched in the lexical tone of the second syllable with the targets. (See Figure 1 for sample stimuli of Experiment 1.) Word frequency and number of strokes were carefully matched across the four printed words in the two conditions (syllabic overlap condition: *Fs* < 1, *ps* > 0.60; sub-syllabic overlap condition: *Fs* < 1, *ps* > 0.82; see Table 1 for the lexical properties of experimental stimuli; word frequency data from the Chinese Linguistic Data Consortium, 2003). All phonological competitors were carefully selected to share no semantic association or orthographic association with target words. Another twenty participants (who did not participate in the following eye-tracking experiment) were recruited and were asked to rate the semantic relatedness between targets and competitors on a 5-point scale. Results showed no significant difference between the two types of competitors (targets and syllabic overlap competitors: *mean score* = 1.15, *SD* = 0.25; targets and sub-syllabic overlap competitors: *mean score* = 1.13, *SD* = 0.21; *t* = 0.45, *p* = 0.65). In addition, two research assistants examined the material to ensure there was no orthographic association between targets and competitors (i.e., sharing no radicals). All critical stimuli were split into two lists. Each list contained 27 syllabic overlap items and 27 sub-syllabic overlap items with no repetition of target words. Each participant was randomly assigned to perform one list only. See the Appendix for all materials used in critical trials.

**TABLE 1:** Lexical properties of the experimental materials in Experiment 1 and 2.

	Target word	Phonological competitor (the syllabic overlap condition)	Phonological competitor (the sub-syllabic overlap condition)	Distractor	Distractor
Mean word frequency	2.39 (2.53)	2.32 (2.61)	2.74 (3.70)	2.20 (2.04)	2.28 (2.11)
Mean number of strokes	16.81 (4.60)	16.94 (4.76)	17.37 (4.19)	17.37 (4.46)	17.81 (4.75)

*Mean word frequency was calculated as occurrences per million. The standard deviants are presented in brackets.*

#### Apparatus

Participants’ eye movements during the experiment were recorded using an EyeLink1000 Desktop tracker (SR Research, Mississauga, ON, Canada), with sampling at a rate of 1000 Hz. The experimental task was programmed using Experimental Builder software (SR Research Ltd). Auditory stimuli were presented to the participant via headphones (Sennheiser, PC 230). Visual stimuli were presented on a 21-inch monitor (resolution: 1024 × 768; refresh rate: 85 Hz) of a Dell computer. The visual stimuli were displayed in black (RGB: 0, 0, 0) against a white background (RGB: 255, 255, 255). Participants were seated about 57 cm away from the display screen and 1 cm on the screen subtended a visual angle of approximately 1°. A chin rest was used to control the participant’s head position. Although the viewing was binocular, only eye movements of the right eye were recorded during the entire procedure.

#### Procedure

Before the experiment, each participant was given a brief introduction to the experiment. The eye-tracker was then calibrated and validated via 9-point calibration prior to the beginning of the experiment. A drift check was performed before the start of each trial. Each trial started with a blank screen displayed for 500 ms, and then followed by a visual display consisting of four printed words. The auditory stimuli were presented 200 ms^3^ after the visual display onset. The four printed words were arranged at the four corners of the screen and the positions of each printed word were randomized across trials^4^. The size of each disyllabic word was approximately 1.5° × 3° and located about 10° away from the screen center. Participants were asked to find the target word (that was same as the auditory stimulus) and click on it using a computer mouse. The visual display remained on the screen until a response was made. Participants were required to make the responses as quickly and accurately as possible.

There were seven practice trials prior to the formal experiment to ensure that participants were familiar with the task procedure. Additional 54 filler trials including a target and three distractors were added into the experiment, which were constructed to avoid participants being aware of the manipulations in the critical trials. Critical trials and filler trials were intermixed and randomly presented. The duration of the whole experiment was approximately 10–15 min.

### Results and Discussion

#### Accuracy Data

Filler trials, practice trials, and critical trials with wrong responses were all excluded from the data analysis. Participants’ accuracy in the critical trials was 99.9%, suggesting that they paid sufficient attention to the task.

#### Data Coding

A square region of 10° × 10°centered around each printed word was designated as the region of interest (ROI) for eye tracking. Only fixations that fell into the ROI were defined as “fixating on the current word” and those fixations that did not fall on the printed words were considered as falling on the background. All fixations were coded as “0” (not fixed) or “1” (fixed) for every 100 ms bin starting from 200 ms before the onset of the auditory stimulus presentation.

#### Eye Movement Data

A logit mixed model (Jaeger, 2008; Quené and van den Bergh, 2008; Ferreira et al., 2013) was employed to analyze the eye movement data in the R software (R Core Team, 2020). A glmer() function in the lme4 package (Version 1.1-23; Bate et al., 2015) was used to build the mixed effect models. For the base model, a random intercept for participants and items was added. Then, the fixed effects (*“the word type”*: competitor vs. distractor and *“the competitor type”*: the syllabic overlap competitor vs. the sub-syllabic overlap competitor) were added into the models one by one, followed by the interaction of the fixed effects as well as the by-participants random slope for the fixed factor (Cunnings, 2012; Barr et al., 2013). To test whether adding a factor improved the model fit, anova() was used to conduct the model comparison. For any significant interaction effect, contrast analysis was performed to compare the effect between the competitor and the distractors.

**FIGURE 2:**
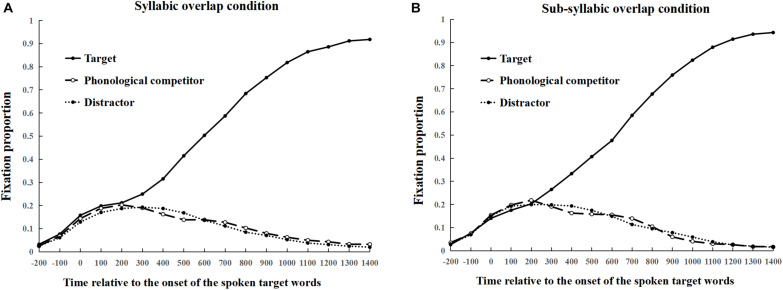
Proportion of fixations on the target, the phonological competitor and the distractors from 200 ms before the onset of the spoken target word in the syllabic overlap condition **(A)**, and sub-syllabic overlap condition **(B)**, respectively in Experiment 1.

Figure 2 presents the proportion of the fixations to the targets, the phonological competitors, and the distractors^5^ in the syllabic overlap and sub-syllabic overlap conditions, respectively. As seen in Figure 2, the fixation proportion curve of the target showed a significant separation trend compared to that of the phonological competitors and distractors after 300 ms from the onset of the spoken words. However, in both conditions, the fixation proportion curves of the phonological competitors and the distractors were almost overlapping except for a very tiny difference in the time window bin of 400–500 ms vs. 500–600 ms. The data for the two time windows were thus analyzed to test whether the difference reached statistical significance. Results of the logit mixed model showed that adding fixed factors or interaction did not improve model fit for either the time window of 400–500 ms [χ*^2^* (2) = 0.24, *p* = 0.89] or 500–600 ms [χ*^2^* (2) = 0.89, *p* = 0.64].

In Experiment 1, no evidence was found for a phonological competition effect on the second syllable (i.e., the rhyme), in agreement with the findings of Malins and Joanisse (2010). This result stands in contrast to the cohort competition effect found in Chinese using a very similar design (Shen et al., 2018). We assume that rhyme processing may be relatively weak compared to cohort processing in Chinese. Therefore, a competitive situation (e.g., presenting a target word in the visual display) may make the subtle rhyme effect less observable. Some previous studies have already shown that the semantic competition effects were larger when the target was not presented in a visual display (i.e., a target-absent design) compared to when the target was presented (i.e., a target-present design) (Huettig and Altmann, 2005). This is because the presence of the target will attract the most attentional resources in the visual field, thus leading to less attention being directed to other visual referents. For this reason, the use of the target-present design may have reduced the likelihood of observing the rhyme competition effect in Experiment 1. Therefore, in Experiment 2, we adopted a target-absent display of the visual-world paradigm to further test the role of the second syllable (i.e., the rhyme) competition effect during Chinese spoken word recognition.

## Experiment 2

### Method

#### Participants

Forty undergraduates (15 men, 25 women) from the same participant pool were randomly recruited to participate in Experiment 2; none of them had participated in Experiment 1. Their ages ranged from 17 to 23 years (*Mean age* = 19.95 years) and they were all native Mandarin Chinese speakers who had normal or corrected-to-normal vision and normal hearing. A small compensation was paid to each participant after the experiment. The research protocol reported here was approved by the university ethics committee.

#### Materials and Apparatus

Materials and apparatus were the same as those used in Experiment 1.

#### Procedure

**FIGURE 3:**
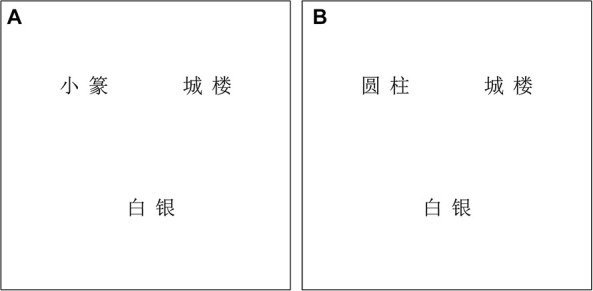
An example of a printed-word display in Experiment 2. For the spoken target word *公转*, *gong1zhuan4*, “revolution,” the printed-word display consisted of a phonological competitor word in the syllabic overlap condition **(A)** *小篆*, *xiao3zhuan4*, “calligraphy” or in the sub-syllabic overlap condition **(B)** *圆柱*, *yuan2zhu4*, “cylinder” and two unrelated distractors *城楼*, *cheng2lou2*, “gate tower” and *白银*, *bai2yin2*, “silver”. In this example, the three printed words were arranged as a V-shape.

The procedure was modified from Experiment 1, in that the visual referents of the spoken target words for the critical trials were not presented on the display screen. Additionally, the spoken target words were presented to participants without the preceding carrier phrase. For each trial, three printed words were presented, arranged as a V-shape or an inverted V-shape on the screen. The positions of the printed words were randomized across trials^6^ (Tsang and Chen, 2010). Each word was located about 8° away from the screen center (see Figure 3 for experimental stimuli of Experiment 2). Participants were presented with spoken target words and instructed to determine whether or not the referent of the spoken target word was on the screen by pressing corresponding buttons on a keyboard. An equivalent number of filler trials were constructed to balance the responses. The referents of spoken targets were presented in the visual display only for filler trials. Thus, “YES” responses were expected in the filler trials, and “NO” responses were expected in the critical trials.

### Results and Discussion

#### Accuracy Data

Filler trials, practice trials, and critical trials with wrong responses were all excluded from the further data analysis. Participants’ accuracy in the critical trials was 99.26%, suggesting that they paid sufficient attention to the task.

#### Eye Movement Data

**FIGURE 4:**
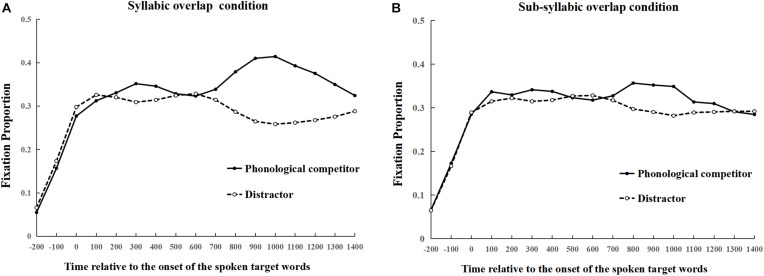
Proportion of fixations on the phonological competitor and the distractors from 200 ms before the onset of the spoken target word in syllabic overlap condition **(A)**, and sub-syllabic overlap condition **(B)**, respectively in Experiment 2.

The data coding and statistical analysis were the same as that in Experiment 1. Figure 4 presents the proportion of the fixations to the phonological competitors and the distractors in the syllabic overlap and sub-syllabic overlap conditions, respectively. The fixation proportion curves of the phonological competitor and the distractor had a clear disassociation from 800 ms until 1300 ms after the onset of the spoken targets. Numerous fixations fell on the phonological competitors compared to the distractors in both conditions. But the degree of divergence in the syllabic overlap condition was larger than that in the sub-syllabic overlap condition. To test whether the differences approached statistical significance, the time windows from 900 to 1300 ms were analyzed using the logit mixed model (see Table 2). The results showed that the model was significantly improved by the interaction of fixed effects and the by-participant random slope of the *“the competitor type”* [χ*^2^* (5) = 16.62, *p* < 0.01] in the time window of 900–1000 ms. The pattern of interaction effects was sustained for the time window from 1000 to 1300 ms (χ*^2^ s* > 8.01, *p*s < 0.05). For the time window of 900–1000 ms, contrast analysis showed that phonological competitors attracted more fixations compared to the distractors in both the syllabic overlap condition (*b* = 0.59, *SE* = 0.09, *Z* = 6.30, *p* < 0.001) and the sub-syllabic overlap condition (*b* = 0.32, *SE* = 0.10, *Z* = 3.41, *p* < 0.001). Moreover, fixation proportions on syllabic overlap competitors were significantly larger than those on the sub-syllabic overlap competitors (*b* = –0.45, *SE* = 0.10, *Z* = –4.59, *p* < 0.001), while no difference was found for the distractors (*Z* = 0.70, *p* = 0.49). The significant phonological competition effects suggested that the second syllable (i.e., the rhyme) was indeed competing and activated during processing of the spoken word perception. More importantly, the phonological activation of sub-syllabic overlap competitors also suggested a significant phonological competition effect of the initial phonemes of the second syllable. In addition, the interaction effects suggested that the activation of phonological competitors was sensitive to phonemic overlapping such that the activation degree of the phonological competitor was varied as a function of the full/partial phonemic overlap. Thus, more visual attention was directed to the syllabic overlap competitors than to the sub-syllabic overlap competitors.

**TABLE 2:** Time windows analysis for results of logit mixed models in the syllabic overlap and sub-syllabic overlap conditions in Experiment 2.

Time window from spoken target word (ms)	Predictor	Syllabic overlap condition				Sub-syllabic overlap condition			
			
		Estimate	Standard error	*Z* values	Corrected *p-*values	Estimate	Standard error	*Z* values	Corrected *p-*values
900–1000 ms	(Intercept)	–1.01	0.07	–14.00	< 0.001***	–1.03	0.07	–13.92	< 0.001***
	Distractor	–0.07	0.10	–0.69	0.49	0.06	0.10	0.63	0.53
	Phonological competitor	0.59	0.09	6.34	< 0.001***	0.32	0.10	3.41	< 0.001***
1000–1100 ms	(Intercept)	–1.29	0.09	–13.91	< 0.001***	–1.28	0.09	–13.71	< 0.001***
	Distractor	0.05	0.10	0.47	0.64	0.10	0.10	0.93	0.35
	Phonological competitor	0.70	0.10	7.10	< 0.001***	0.33	0.10	3.17	< 0.001***
1100–1200 ms	(Intercept)	–1.57	0.11	–13.96	< 0.001***	–1.54	0.12	–13.10	< 0.001***
	Distractor	0.16	0.11	1.44	0.15	0.10	0.11	0.95	0.34
	Phonological competitor	0.62	0.11	5.88	< 0.001***	0.17	0.11	1.54	0.12
1200–1300 ms	(Intercept)	–1.71	0.14	–12.61	< 0.001***	–1.85	0.14	–13.19	< 0.001***
	Distractor	–0.19	0.12	–1.58	0.11	0.07	0.12	0.55	0.58
	Phonological competitor	0.31	0.11	2.76	< 0.01**	0.09	0.12	0.79	0.43

**p < 0.01, ***p < 0.001.

Unexpectedly, we also found a main effect of *“the word type”* in the time window of 300–500 ms. The fixation proportions on phonological competitors were significantly higher than those on distractors in both conditions (χ*^2^*s > 6.86, *p*s < 0.04). However, no interaction of fixed effects was found (χ*^2^*s < 1, *ps* > 0.70). We assume that the mismatched word frequency for some items may account for this unexpected result. After a careful check of the material, we found that the word frequency of item 28 did not match perfectly across conditions such that the word frequency of the sub-syllabic overlap competitor (i.e., *“爆炸,” bao4zha4*, *Word frequency* = 23.31 occurrences per million) was much higher than the word frequencies of the distractors (i.e., *“陵墓,” ling2mu4*, *Word frequency* = 2.14 occurrences per million, *“座椅,” zuo4yi3*, *Word frequency* = 2.19 occurrences per million). When a further data analysis excluding item 28 was conducted, results showed that the unexpected main effect in the time window of 300–500 ms disappeared (χ*^2^ s* > 3.45, *p*s > 0.13), and our main interaction effects in the time windows of 900–1300 ms remained significant (χ*^2^ s* > 6.91, *p*s < 0.05). In addition, the mean word frequency and number of strokes between targets and the competitors and distractors remained matched (syllabic overlap condition: *Fs* < 1, *ps* > 0.69; sub-syllabic overlap condition: *Fs* < 1, *ps* > 0.54)^7^.

## General Discussion

Two experiments were conducted to determine whether the second syllable compete for word recognition and whether the initial-part of the second syllable is involved in the processing of Chinese disyllabic word recognition using a printed-word version of the visual-world paradigm. We manipulated the competitors sharing full phonemes of the second syllable with the targets (i.e., “the syllabic overlap condition”) or sharing the cohort-part phonemes of the second syllable with the targets (i.e., “the sub-syllabic overlap condition”). No main effect or any interaction effect was found to be significant when the targets were presented in the visual display in Experiment 1. However, a significant phonological competition effect was observed under both conditions when the target referents were removed from the visual display in Experiment 2 (i.e., the fixation proportions on the phonological competitors were higher than those on distractors) in both syllabic overlap and sub-syllabic overlap conditions, suggesting that both second syllable (i.e., the rhyme) and the initial part of the second syllable were accessed for competition. In addition, we also found a larger competition effect in the syllabic overlap condition than that in the sub-syllabic overlap condition in Experiment 2.

In the current study, we observed clear and robust evidence that the second syllable (which was usually deemed as a rhyme in previous Chinese studies, Liu et al., 2006; Meng, 2014) are involved in competition in Chinese spoken disyllabic word recognition using a target-absent display of the visual-world paradigm. Prior studies regarding rhyme processing in Chinese have yielded contradictory results. Some studies have found no evidence for rhyme competition while other studies have observed a rhyme competition effect (Zhao et al., 2011; Meng, 2014). To revisit this issue and maximize the possibility of observing a compound rhyme competition effect on disyllabic words, we altered some aspects of our original experimental design, i.e., presenting only one competitor type and presenting targets in isolation, using the printed-word paradigm. The results showed an important role of the second syllable (i.e., the rhyme) in Chinese spoken word recognition, in that the syllabic overlap competitors attracted more fixations than did the distractors. The significant rhyme effect in Chinese is consistent with the role of rhyme for disyllabic words in alphabetical languages, showing a universal competitive role of rhymes across languages. However, the rhyme competition effect in Chinese was only observed in the target-absent situation in Experiment 2 and the effect disappeared in the target-present situation in Experiment 1. These results seem to indicate that the rhyme effect for disyllabic words in Chinese is relatively weak, such that a competitive environment (such as presenting targets on a screen) weakens the rhyme effect to the point of producing a null effect in Experiment 1.

More importantly, we also observed the activation of the initial phonemes of the second syllable (i.e., rhyme) during spoken word recognition. As noted, most studies focused on the separate role of cohort/rhyme and few studies have ever investigated the role of embedded cohort in the rhyme. The significant competition effect observed in the sub-syllabic overlap condition suggested that the embedded cohort part within a rhyme was also activated and the rhyme processing in the disyllabic word was accessed in a multiple-layer way: the whole-word layer and the monosyllabic layer. In addition, we also observed a modulating role of phonemic overlapping proportion on the activation degree of the phonological competitors; a larger competition effect was found for syllabic overlap competitors and a relatively small competition effect was found for sub-syllabic overlap competitors, demonstrating that the mapping of spoken signals onto the inner lexical representation was determined by the degree of phonological similarity. Therefore, syllabic overlap competitors with full phoneme overlapping with targets are activated to a higher degree than the sub-syllabic overlap competitors with only partial phoneme overlapping with targets. This is reflected in eye movement behaviors as more fixations are directed to the syllabic overlap competitors than to the sub-syllabic overlap competitors.

Given that the second syllable was considered as the rhyme in prior Chinese disyllabic word processing (Liu et al., 2006; Meng, 2014), the current findings also contribute to refinements of the rhyme processing assumptions in the spoken word recognition models. Existing spoken word recognition models make different assumptions regarding rhyme processing. The second syllable (i.e., rhyme) competition effect observed in the present study is in line with the assumptions of the TRACE model (McClelland and Elman, 1986) which assumes that spoken word recognition is a continuous process with the competition among rhymes being involved. The TRACE model also predicts that rhymes and cohorts are processed with different weights, with a weaker and later activation of the rhyme processing but an earlier and stronger activation for the cohort processing. Our current finding of an absence of a rhyme competition effect in the target-present situation stands in contrast to the significant cohort effect found in the target-present situation in our prior studies (Shen et al., 2018). The combined results across two studies provide some indication that the rhyme effect is relatively weaker than the cohort effect in Chinese. In addition, both the TRACE and TTRACE model (proposed by Tong et al., 2014) make no assumptions about how the embedded cohort/rhyme are processed in Chinese spoken word recognition. Our current findings provided the first piece of evidence that the embedded cohort part of the rhyme can also be activated during Chinese disyllabic word recognition. The significant phonological competition effect observed in the sub-syllabic overlap condition in Experiment 2 also shed some lights on the uniqueness and complication of the rhyme processing in Chinese. The rhyme processing in Chinese is likely to be accessed in a multiple-layer manner: the whole word layer and the monosyllabic layer, and the rhymes are possibly to be accessed in parallel or serially at these two different levels.

It is well known that compared to alphabetical languages such as English, most syllables in Chinese can be mapped onto morphemes and can stand alone as monosyllabic words (Zhou and Marslen-Wilson, 1994; Zhao et al., 2011). This specific linguistic property may lead to different rhyme representations in Chinese (especially in disyllabic Chinese words) as compared to that in alphabetical languages such that rhyme representation in Chinese may be more salient than that in alphabetical languages. However, the saliency of rhyme representation in Chinese may be affected by word frequency. Prior studies have found that low-frequency words are more likely to be represented as separated morphemic entries while high-frequency words are more likely to be represented as whole-word entries in the mental lexicon (Pollatsek et al., 2000; Yan et al., 2006). Thus, it is possible that the separated morphemic representations of low-frequency words may also increase the saliency of the linking phonological representations (i.e., rhyme representation) compared to high-frequency words. In our current study, the mean word frequency of the target words is 2.39 occurrences per million. Based on a Chinese Linguistic Data Consortium (2003), only 22.86% words’ frequencies are higher than the targets. Therefore, target words in current study are relatively high-frequency Chinese words and the rhyme representations of those words are not that salient. In this study, we did not manipulate the word frequency of targets directly, and thus it remains unclear how the word frequency would influence rhyme processing and representations. More studies need to be conducted to further examine this issue.

One may argue that the phonological competition effect observed in the target-absent design in Experiment 2 may have resulted from task-specific strategies. For example, participants may allocate more attentional resources to the target absent displays because the task was to search for a matched target. However, this explanation is less plausible because: (1) the visual display was previewed for 200 ms before the onset of the auditory words. Given that 200 ms is usually assumed to be the retrieval time for phonological information from printed words (Huettig and McQueen, 2007), this preview time prevented listeners from searching for visual words strategically and accessing the phonological code of printed words based on the phonological information of the spoken targets. The setting of the preview time allows that the phonological information of the spoken word and the printed words was accessed concurrently and the eye movement measures of the printed words reflected the ongoing cognitive processing during spoken word recognition rather than a later search effect after the target words had been activated. (2) In addition, participants in the current study were instructed to search for a target word in the visual display and responded by pressing keys. It should be noted that no explicit phonological processing was necessary in order to complete the task. If the phonological competition effect had resulted from a visual search strategy, then the fixation proportion under both syllabic overlap and sub-syllabic overlap conditions should be the same since both the syllabic overlap and sub-syllabic overlap competitors were not targets and there was no reason for participants’ eyes to fixate on these visual referents. However, the significant competition effect suggested that the phonological information of the second syllable was indeed activated. Based on this fact, we argue that the phonological information of the second syllable was activated automatically to a larger extent during the visual search and less likely to have been the result of the task. More future studies need to be conducted to further examine whether the phonological competition effect on rhymes and on partial phonemes of rhymes still exist under a more general and natural language processing situation.

Our study has several limitations worth noting. First, the lexical tone of the phonological competitors was not manipulated in the current study. One of our prior studies showed that lexical tone affects the degree of activation of cohort competitors (Shen et al., 2020), and thus it remains unclear how the lexical tone of rhymes might modulate the activation degree of rhyme competitors. This should be investigated in future studies. Second, the current study did not include a cohort competitor type in the word level, and thus it was unable to directly compare the processing differences in time course and activation degree between the two types of phonological candidates on same level during spoken word recognition. Third, in the current study, we only considered the processing of word-initial phonemes (i.e., cohort-part) in the syllable level (i.e., second syllable). Future studies need to be conducted to further explore whether the word-final phonemes (i.e., rhyme-part) of the second syllable can also be activated. These studies have important implications in uncovering the uniqueness of rhyme processing in Chinese. Fourth, current study did not intentionally control or manipulate the participants’ cognitive/meta-linguistic abilities or the familiarity degree of Chinese printed words. It is possible that those factors may also exert some confounding influence on our current findings. Future studies need to be conducted to further investigate the role of these factors on the syllable processing in Chinese. Lastly, more future studies need to be designed to investigate how context predictability may influence rhyme processing in Chinese spoken perception.

Taken together, the current study confirmed that not only the second syllable (i.e., rhyme) at the word level is activated, but also the initial phonemes of the second syllable at the syllabic level are also activated for competition in Chinese spoken disyllabic word recognition.

## Data Availability Statement

The original contributions presented in the study are included in the article/supplementary material, further inquiries can be directed to the corresponding author via shen_wei@yahoo.com.

## Ethics Statement

The studies involving human participants were reviewed and approved by the Ethics Committee of the Institute of Psychological Sciences from Hangzhou Normal University. The participants provided their written informed consent to participate in this study.

## Author Contributions

YW and WS conceived and designed the experiments. YW and HZ performed the experiments. YW and WS analyzed the data. YW, XZ, and WS wrote the manuscript. All the authors contributed to the article and approved the submitted version.

## Conflict of Interest

The authors declare that the research was conducted in the absence of any commercial or financial relationships that could be construed as a potential conflict of interest.

## Publisher’s Note

All claims expressed in this article are solely those of the authors and do not necessarily represent those of their affiliated organizations, or those of the publisher, the editors and the reviewers. Any product that may be evaluated in this article, or claim that may be made by its manufacturer, is not guaranteed or endorsed by the publisher.

## Footnotes

1 In previous alphabetical languages studies, rhymes were defined as sharing similar ending phonemes in the final syllable between two or more words (e.g., *“beaker”* – *“speaker”* in Allopenna et al., 1998). In Chinese disyllabic words studies, rhymes were defined by the second syllable (e.g., *桥洞, qiao2dong4*, “arches,” – *果冻, guo3dong4*, “jelly” in Meng, 2014; *电池, dian4chi2*, “battery”- *水池, shui3chi2*, “water pool” in Liu et al., 2006). Similarly, in the current study, we also considered the second syllable to be the shared rhyme between the target and competitor words.

2 Chinese characters of the examples in Malins and Joanisse (2010) were not given in the original article. We added those Chinese characters to ease the understanding for readers.

3 This choice of preview time prevents participants from reading the words strategically.

4 For each spoken target word, the positions of printed targets, competitors, and distractors were the same in the syllabic overlap and sub-syllabic overlap conditions.

5 The curve of distractors refers to the average fixation proportions of the two distractors in Figures 2, 4.

6 For each spoken target word, the positions of competitors and distractors were the same in the syllabic overlap and sub-syllabic overlap conditions.

7 Mean word frequency: syllabic overlap competitors, *M* = 2.36, *SD* = 2.62; sub-syllabic overlap competitors, *M* = 2.35, *SD* = 2.38; distractor, *M* = 2.20, *SD* = 2.06; distractor, *M* = 2.28, *SD* = 2.13; Mean number of strokes: syllabic overlap competitors, *M* = 16.94, *SD* = 4.81; sub-syllabic overlap competitors, *M* = 17.17, *SD* = 3.96; distractor, *M* = 17.26, *SD* = 4.43; distractor, *M* = 17.74, *SD* = 4.76.

## Appendix

**TABLE A1:** Materials used in Experiments 1 and 2.

Id	Target	Phonological competitor (Syllabic overlap condition)	Phonological competitor (Sub-syllabic overlap condition)	Distractor	Distractor
1	电铃 (dian4 ling2)	幽灵 (you1 ling2)	山林 (shan1 lin2)	房东 (fang2 dong1)	默契 (mo4 qi4)
2	脾脏 (pi2 zang4)	土葬 (tu3 zang4)	短暂 (duan3 zan4)	嫩叶 (nen4 ye4)	麦穗 (mai4 sui4)
3	山羊 (shan1 yang2)	洛阳 (luo4 yang2)	感言 (gan3 yan2)	体操 (ti3 cao1)	媒介 (mei2 jie4)
4	豆浆 (dou4 jiang1)	边疆 (bian1 jiang1)	管家 (guan3 jia1)	温室 (wen1 shi4)	孔雀 (kong3 que4)
5	纸张 (zhi3 zhang1)	图章 (tu2 zhang1)	残渣 (can2 zha1)	水壶 (shui3 hu2)	祖辈 (zu3 bei4)
6	旧账 (jiu4 zhang4)	方丈 (fang1 zhang4)	油炸 (you2 zha4)	星空 (xing1 kong1)	拖鞋 (tuo1 xie2)
7	公转 (gong1 zhuan4)	小篆 (xiao3 zhuan4)	圆柱 (yuan2 zhu4)	城楼 (cheng2 lou2)	白银 (bai2 yin2)
8	浑浊 (hun2 zhuo2)	烧灼 (shao1 zhuo2)	翠竹 (cui4 zhu2)	情义 (qing2 yi4)	泰斗 (tai4 dou3)
9	阴阳 (yin1 yang2)	绵羊 (mian2 yang2)	精盐 (jing1 yan2)	自卑 (zi4 bei1)	气球 (qi4 qiu2)
10	臭氧 (chou4 yang3)	瞻仰 (zhan1 yang3)	眉眼 (mei2 yan3)	内科 (nei4 ke1)	绳索 (sheng2 suo3)
11	精英 (jing1 ying1)	妇婴 (fu4 ying1)	高音 (gao1 yin1)	圣旨 (sheng4 zhi3)	电车 (dian4 che1)
12	军营 (jun1 ying2)	轻盈 (qing1 ying2)	水银 (shui3 yin2)	曲调 (qu3 diao4)	早餐 (zao3 can1)
13	纸箱 (zhi3 xiang1)	丁香 (ding1 xiang1)	对虾 (dui4 xia1)	阴间 (yin1 jian1)	跳蚤 (tiao4 zao3)
14	母性 (mu3 xing4)	荣幸 (rong2 xing4)	书信 (shu1 xin4)	脸蛋 (lian3 dan4)	电量 (dian4 liang4)
15	兴旺 (xing1 wang4)	遗忘 (yi2 wang4)	手腕 (shou3 wan4)	奥秘 (ao4 mi4)	交警 (jiao1 jing3)
16	轻伤 (qing1 shang1)	智商 (zhi4 shang1)	乌纱 (wu1 sha1)	毒瘤 (du2 liu2)	独白 (du2 bai2)
17	松香 (song1 xiang1)	梦乡 (meng4 xiang1)	龙虾 (long2 xia1)	卷发 (juan3 fa4)	野营 (ye3 ying2)
18	双脚 (shuang1 jiao3)	直角 (zhi2 jiao3)	马甲 (ma3 jia3)	恩爱 (en1 ai4)	火海 (huo3 hai3)
19	辣酱 (la4 jiang4)	锁匠 (suo3 jiang4)	年假 (nian2 jia4)	巫女 (wu1 nv3)	戏服 (xi4 fu2)
20	王冠 (wang2 guan1)	双关 (shuang1 guan1)	地瓜 (di4 gua1)	舅妈 (jiu4 ma1)	孩童 (hai2 tong2)
21	芦荡 (lu2 dang4)	文档 (wen2 dang4)	鸭蛋 (ya1 dan4)	恶梦 (e4 meng4)	衣架 (yi1 jia4)
22	朝纲 (chao2 gang1)	水缸 (shui3 gang1)	猪肝 (zhu1 gan1)	牙医 (ya2 yi1)	逆贼 (ni4 zei2)
23	干冰 (gan1 bing1)	败兵 (bai4 bing1)	礼宾 (li3 bin1)	玉佩 (yu4 pei4)	黑锅 (hei1 guo1)
24	佚名 (yi4 ming2)	神明 (shen2 ming2)	烟民 (yan1 min2)	街区 (jie1 qu1)	水盆 (shui3 pen2)
25	车篷 (che1 peng2)	暖棚 (nuan3 peng2)	瓦盆 (wa3 pen2)	腮红 (sai1 hong2)	柔光 (rou2 guang1)
26	乳糖 (ru3 tang2)	澡堂 (zao3 tang2)	酒坛 (jiu3 tan2)	稀泥 (xi1 ni2)	幼芽 (you4 ya2)
27	科幻 (ke1 huan4)	祸患 (huo4 huan4)	白桦 (bai2 hua4)	桌布 (zhuo1 bu4)	街巷 (jie1 xiang4)
28	胜仗 (sheng4 zhang4)	结账 (jie2 zhang4)	爆炸 (bao4 zha4)	陵墓 (ling2 mu4)	座椅 (zuo4 yi3)
29	肾脏 (shen4 zang4)	宝藏 (bao3 zang4)	夸赞 (kua1 zan4)	美梦 (mei3 meng4)	红日 (hong2 ri4)
30	弹簧 (tan2 huang2)	女皇 (nv3 huang2)	精华 (jing1 hua2)	油菜 (you2 cai4)	旋风 (xuan2 feng1)
31	春风 (chun1 feng1)	先锋 (xian1 feng1)	高分 (gao1 fen1)	嘴巴 (zui3 ba1)	机密 (ji1 mi4)
32	勋章 (xun1 zhang1)	纸张 (zhi3 zhang1)	山楂 (shan1 zha1)	美景 (mei3 jing3)	幽灵 (you1 ling2)
33	宝剑 (bao3 jian4)	组件 (zu3 jian4)	暑假 (shu3 jia4)	群落 (qun2 luo4)	指纹 (zhi3 wen2)
34	界限 (jie4 xian4)	电线 (dian4 xian4)	属下 (shu3 xia4)	老虎 (lao3 hu3)	滋味 (zi1 wei4)
35	元帅 (yuan2 shuai4)	蟋蟀 (xi1 shuai4)	票数 (piao4 shu4)	情侣 (qing2 lv3)	下肢 (xia4 zhi1)
36	驾照 (jia4 zhao4)	口罩 (kou3 zhao4)	奸诈 (jian1 zha4)	蚊虫 (wen2 chong2)	灾荒 (zai1 huang1)
37	香肠 (xiang1 chang2)	特长 (te4 chang2)	巡察 (xun2 cha2)	粉笔 (fen3 bi3)	丝巾 (si1 jin1)
38	主线 (zhu3 xian4)	权限 (quan2 xian4)	惊吓 (jing1 xia4)	明月 (ming2 yue4)	物料 (wu4 liao4)
39	失明 (shi1 ming2)	芳名 (fang1 ming2)	灾民 (zai1 min2)	烤鸭 (kao3 ya1)	油污 (you2 wu1)
40	肉馅 (rou4 xian4)	眼线 (yan3 xian4)	初夏 (chu1 xia4)	宝刀 (bao3 dao1)	徽章 (hui1 zhang1)
41	棒冰 (bang4 bing1)	逃兵 (tao2 bing1)	来宾 (lai2 bin1)	面皮 (mian4 pi2)	家眷 (jia1 juan4)
42	行程 (xing2 cheng2)	商城 (shang1 cheng2)	清晨 (qing1 chen2)	古董 (gu3 dong3)	枝叶 (zhi1 ye4)
43	圣灵 (sheng4 ling2)	欺凌 (qi1 ling2)	松林 (song1 lin2)	长辈 (zhang3 bei4)	操场 (cao1 chang3)
44	圆锥 (yuan2 zhui1)	尾追 (wei3 zhui1)	植株 (zhi2 zhu1)	青蛙 (qing1 wa1)	佳肴 (jia1 yao2)
45	情商 (qing2 shang1)	枪伤 (qiang1 shang1)	细砂 (xi4 sha1)	问卷 (wen4 juan3)	媒婆 (mei2 po2)
46	藏青 (zang4 qing1)	左倾 (zuo3 qing1)	单亲 (dan1 qin1)	指环 (zhi3 huan2)	假牙 (jia3 ya2)
47	水缸 (shui3 gang1)	朝纲 (chao2 gang1)	标杆 (biao1 gan1)	酒窝 (jiu3 wo1)	脑瘤 (nao3 liu2)
48	赏封 (shang3 feng1)	险峰 (xian3 feng1)	秋分 (qiu1 fen1)	灵位 (ling2 wei4)	厨艺 (chu2 yi4)
49	公关 (gong1 guan1)	外观 (wai4 guan1)	西瓜 (xi1 gua1)	热量 (re4 liang4)	昆虫 (kun1 chong2)
50	生姜 (sheng1 jiang1)	九江 (jiu3 jiang1)	亲家 (qing4 jia1)	北欧 (bei3 ou1)	富豪 (fu4 hao2)
51	特效 (te4 xiao4)	欢笑 (huan1 xiao4)	盛夏 (sheng4 xia4)	踪影 (zong1 ying3)	陵墓 (ling2 mu4)
52	酒精 (jiu3 jing1)	水晶 (shui3 jing1)	钢筋 (gang1 jin1)	植被 (zhi2 bei4)	舌头 (she2 tou)
53	蛛网 (zhu1 wang3)	来往 (lai2 wang3)	饭碗 (fan4 wan3)	水池 (shui3 chi2)	品格 (pin3 ge2)
54	证件 (zheng4 jian4)	弓箭 (gong1 jian4)	书架 (shu1 jia4)	月饼 (yue4 bing3)	明珠 (ming2 zhu1)

*Words’ pronunciations are presented in brackets.*
